# The Assessment of Upper Airway Volume Changes Following Bimaxillary Advancement Surgery: A Radiological Evaluation in the Supine Position at Multiple Intervals

**DOI:** 10.3390/jcm13164588

**Published:** 2024-08-06

**Authors:** Paweł Piotr Grab, Michał Szałwiński, Maciej Jagielak, Jacek Rożko, Dariusz Jurkiewicz, Aldona Chloupek, Maria Sobol, Piotr Rot

**Affiliations:** 1 Clinical Department of Cranio-Maxillo-Facial Surgery, Military Institute of Medicine—National Research Institute, Szaserów 128, 04-141 Warsaw, Poland; mszalwinski@wim.mil.pl (M.S.); jrozko@wim.mil.pl (J.R.); achloupek@wim.mil.pl (A.C.); 2 Department of Dental and Maxillofacial Radiology, Medical University of Warsaw, 02-097 Warsaw, Poland; 3 Ortognatyka Dr Jagielak—Private Surgical Practice, Al. Krakowska 54, 05-090 Raszyn, Poland; mjagielak@ortognatyka.pl; 4 Clinical Department of Otolaryngology, Military Institute of Medicine—National Research Institute, Szaserów 128, 04-141 Warsaw, Poland; djurkiewicz@wim.mil.pl (D.J.); prot@wim.mil.pl (P.R.); 5 Department of Biophysics, Physiology and Pathophysiology, Medical University of Warsaw, 02-901 Warsaw, Poland; maria.sobol@wum.edu.pl

**Keywords:** orthognathic surgery, upper airway, bimaxillary surgery, obstructive sleep apnea, postoperative, CT scan

## Abstract

**Background**: Bimaxillary surgeries (BiMax) are an essential part of the craniomaxillofacial specialty. The osteotomies and subsequent spatial rearrangement of the maxilla and the mandible enable the correction of facial deformities, asymmetry, and malocclusion. Moreover, the movements performed during the procedure affect the morphology of surrounding soft tissues, including the upper airway (UA). **Objectives:** The objective of this study was to radiologically assess the potential volumetric alterations of the UA in the supine position at various intervals following BiMax advancement surgeries. **Methods**: A group of 31 patients who underwent BiMax advancement surgery were included in the study. Medical computed tomography (CT) of the head and neck region was performed 2 weeks preoperatively, 1 day postoperatively, and 6 months postoperatively. The UA volumes were calculated and analyzed based on the acquired Digital Imaging and Communications in Medicine (DICOM) files using different software applications. The sella-nasion-A point (SNA) and sella-nasion-B point (SNB) angles were evaluated to measure the achieved maxillomandibular advancement. **Results**: When comparing the volume of the UA before surgery, post-surgery, and 6 months post-surgery, the *p*-value was <0.001, indicating statistically significant differences in UA volume between the successive examinations. A statistically significant difference was found between UA volume before surgery and 6 months post-surgery and between UA volume after surgery and 6 months post-surgery, with the obtained *p*-values being <0.001 and 0.001, respectively. A significantly larger UA volume was observed 6 months post-surgery (mean ± SD: 27.3 ± 7.3) compared to the volume before surgery (mean ± SD: 22.2 ± 6.4), as well as 6 months post-surgery compared to the volume assessed shortly after surgery (mean ± SD: 24.2 ± 7.3). **Conclusions**: BiMax advancement surgeries result in the significant enlargement of the UA. The volume of the UA does not diminish immediately following the procedure and is not constant; it increases significantly during the postoperative observation period.

## 1. Introduction

Bimaxillary surgery (BiMax) is a cornerstone technique of orthognathic surgery, a fundamental area within the craniomaxillofacial specialty. It involves precise osteotomies of the maxilla–LeFort I osteotomy and the mandible–bilateral sagittal split osteotomy (BSSO), with the proper mobilization and spatial rearrangement of the osteotomized bones. When the maxilla and the mandible are moved forward in a sagittal plane, the BiMax procedure is called an advancement. It can be achieved both with angular and/or linear movements [1]. One of the key aspects of the preparation for orthognathic surgery is proper planning. Currently, methods of virtual planning and 3-D printing are being used more and more frequently in that process [2].

The primary applications for BiMax advancement surgery are malocclusion resulting from disproportions of the maxillomandibular complex, facial asymmetry, spatial discrepancies of the mid- and lower face, and coexisting temporomandibular joint dysfunctions. Another significant clinical indication for the procedure is the presence of obstructive sleep apnea (OSA) stemming from the limited volume of the patient’s upper airway (UA). With appropriate qualifications, the procedure can improve masticatory function, vocalization, and breathing, as well as the patients’ psychological well-being and self-esteem [1,3,4].

The osteotomy of the maxilla is performed mostly along the LeFort I line. It has been used since the 1860s, primarily to correct a wide range of facial skeleton malformations. During the procedure, the maxilla is accurately cut, disimpacted, and fully mobilized. In some cases, the maxilla can be further segmented to achieve adequate functional occlusion [5].

The BSSO consists of bilateral osteotomies of the mandible, which can be performed with the abundance of modifications provided by Trauner and Obwegeser, Epcker, Wolford, and Dal Pont et al. The unifying element of the procedure is the sagittal plane of the cut and the preservation of the inferior alveolar nerve connected to the distal mandibular segment only [6].

The BiMax can be a maxilla- or mandible-first procedure, based on the specific case and the preference of the surgeon. Both the maxillary and the mandibular osteotomized segments are spatially repositioned and stabilized in accordance with the surgical plan. A relatively high level of accuracy in the procedure can be achieved thanks to the 3D-printed intermaxillary splints [2,7].

The airway is a component of the respiratory system that can be anatomically divided into several sections. The upper part of it, beginning with the epiglottis, is functionally and morphologically related to the bones and muscles, which are altered during orthognathic procedures. Patients who are subjects of BiMax advancement procedures often present with extensive craniofacial defects, partially resulting from improper jaw growth in the sagittal plane. With respect to those deformities, they often result in a decrease in the UA volume and, thus, may increase the incidence of obstructive sleep apnea (OSA) [8,9].

The purpose of this study is to evaluate the volume changes in the UA resulting from BiMax advancement surgeries. This research is based on the classic computed tomography (CT) data acquired in the supine position of the patients. We hypothesize that BiMax advancement results in an increase in the volume of the UA, both in short- and long-term evaluations, and that the achieved immediate values change postoperatively.

## 2. Materials and Methods

### 2.1. Eligibility Requirements

The inclusion criteria are the following: adults (over 18 years old) who underwent BiMax advancement surgery in our department; patients presenting with either skeletal class II or class III malocclusion preoperatively; CT examinations performed in our department according to a regimen of specific time points; and preoperative decompensatory treatment using fixed orthodontic appliances.

The exclusion criteria are the following: minors (age below 18 years old); prior history of any medical incidents or procedures of the upper respiratory tract, e.g., tonsillectomy, pharyngoplasty, and soft palate plasty; revision orthognathic surgery; and non-compliance with follow-up appointments or postoperative orthodontic treatment.

### 2.2. Treatment

All patients included in the study underwent BiMax advancement procedures performed by the same surgical team in the Cranio-Maxillo-Facial department of the Military Institute of Medicine, Warsaw, Poland, between 1 January 2022 and 31 December 2023. (P.G.; J.R.; and M.S.). The mandible-first approach was utilized in each case. The surgeries were virtually planned using IPS CASE DESIGNER^®^ software, v2.5.7.1 (KLS Martin Group, Tuttlingen, Germany), with both jaws’ advancement and maxillary impaction of less than 4 mm. The intermediate and final virtual surgical splints were exported as an STL. file and 3D printed with a medical-grade, vat photopolymerization printing machine (Next Dent 5100; Next Dent, Soesterberg, The Netherlands) using surgical guide resin (Next Dent SG, Next Dent, Soesterberg, The Netherlands). None of the cases involved bone grafting. The osteotomies were executed using piezo surgery equipment and standard bone chisels. Each patient was provided with identical titanium osteosynthesis hardware, system 2.0 miniplates, and screws from KLS Martin (KLS Martin Group, Tuttlingen, Germany).

### 2.3. Data Acquisition

All the patients included in the study underwent three non-contrast CT scans of the head and neck region at specified time points: 2 weeks preoperatively, 1 day postoperatively, and 6 months postoperatively. CT was performed in a supine position, with the patient instructed to remain in current occlusion with the condition of not performing swallowing actions during the examination. Radiological data were acquired in a Digital Imaging and Communications in Medicine (DICOM) format with a 0.6 mm slice thickness. The same 64-slice CT scanner was used consistently in all cases.

### 2.4. Measurements

**Figure 1:**
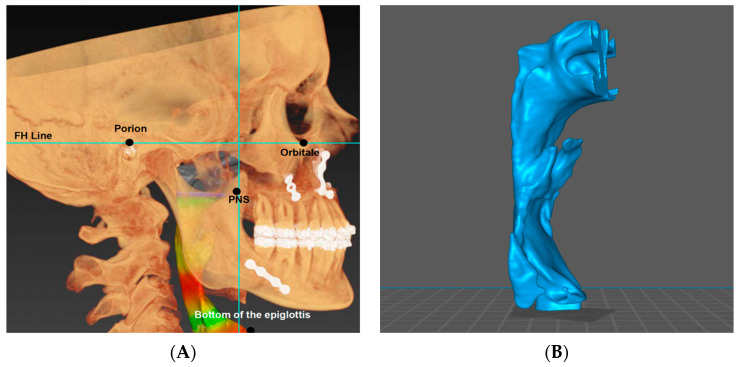
(**A**) Cephalometric skeletal landmarks with UA reconstruction; (**B**) 3D visualization of the UA analyzed according to the provided protocol.

All the measurements and analyses were performed by P.G. and J.R. The collected DICOM data were initially analyzed in the IPS CASE DESIGNER^®^ software 2.5.7.1. Three-dimensional virtual craniofacial models were superimposed based on the cephalometric points and lines: Orbitale, Nasion, Basion, Porion, and the Frankfurt Plane (FH plane). The planned advancement of both jaws resulting from the surgery was cephalometrically confirmed in all cases. The sella-nasion-A point (SNA) and sella-nasion-B point (SNB) angles, which respectively denote the anteroposterior position of the maxilla and the mandible relative to the cranial structures, were evaluated individually before surgery and 6 months postoperatively. The volumetric examination of the UA was calculated with the following limits (Figure 1A,B):

- Anterior boundary—plane perpendicular to the FH plane passing through the Posterior Nasal Spine point (PNS).
- Superior boundary—the highest point of the nasopharynx.
- Inferior boundary—plane perpendicular to the FH plane passing through the bottom of the epiglottis.

The volume of the acquired 3D UA models was evaluated and cross-checked between the IPS CASE DESIGNER^®^ software and the BLENDER^®^ software, v4.0 (Blender Foundation, Amsterdam, The Netherlands) to assure the validity of the presented measures.

### 2.5. Statistical Analysis

The statistical analysis was performed using Statistica 13.3 by Dell Software Inc. (Round Rock, TX, USA). For the cohort of 31 patients, the statistical power to detect significant differences between preoperative and 6-month postoperative outcomes at α = 0.05 was determined to be 78%.

Descriptive statistics are presented as means ± SD, median, range, and interquartile range (IQR). The conformity of the distribution of quantitative variables to the normal distribution was checked using the Shapiro–Wilk test.

Since the distribution of the variables conformed to the normal distribution, an ANOVA with repeated measures was used to determine whether there were statistically significant differences in the volume of the upper airways before the procedure, after the procedure, and 6 months post-procedure. The level of statistical significance was set at α = 0.05. Additionally, to check for statistically significant differences between pairs of visits, a paired *t*-test was used. Due to multiple comparisons, Bonferroni corrections were applied, and the level of significance was set at 0.017.

## 3. Results

A total of 31 patients participated in the study. The age distribution of the group was: mean ± SD: 28.35 ± 6.83; median: 27; range: 18 to 48. Of the 31 participants in the study, 24 were women and 7 were men. All participants were Caucasian. The group included 22 patients with skeletal class III malocclusion and 9 patients with skeletal class II malocclusion (Table S1; Table 1). The distribution between skeletal classes II and III is solely due to the group of patients who presented to our clinic during the specified time and met the inclusion and exclusion criteria for the study.

The mean ± SD SNA angle value increased from 79.89 ± 3.69 to 84.79 ± 4.04 degrees, and the mean ± SD SNB angle value increased from 80.55 ± 4.74 to 82.47 ± 4.21 degrees.

**Figure 2:**
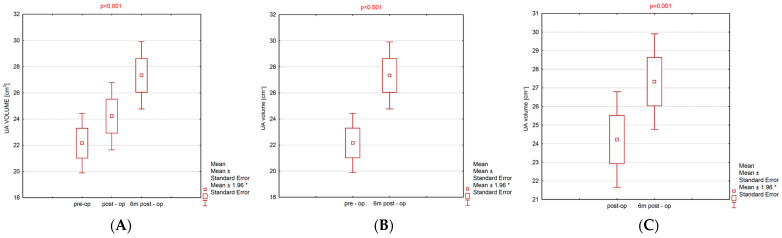
Mean; Mean ± Standard Error; Mean ± 1.96 * Standard Error and *p*-value diagrams. (**A**) Pre-op vs. post-op vs. 6 m post-op. (**B**) Pre-op vs. 6 m post-op. (**C**) Post-op vs. 6 m post-op.

When comparing the volume of the UA before surgery, post-surgery, and 6 months post-surgery, the obtained *p*-value was <0.001, indicating statistically significant differences in UA volume between the successive examinations. After applying the paired *t*-test, statistically significant differences were found between UA volume before surgery and 6 months post-surgery, and between UA volume after surgery and 6 months post-surgery, with the obtained p-values being <0.001 and 0.001, respectively. A significantly larger UA volume was observed 6 months post-surgery (mean ± SD: 27.3 ± 7.3) compared to the volume before the surgery (mean ± SD: 22.2 ± 6.4), as well as 6 months post-surgery compared to the volume assessed shortly after the surgery (mean ± SD: 24.2 ± 7.3). (Table S1; Table 2; Table 3; Figure 2A–C).

The mean UA volume in a first postsurgical examination was larger (mean ± SD: 24.2 ± 7.3) compared to the volume before the surgery (mean ± SD: 22.2 ± 6.4), but this result was not statistically significant. (Table S1; Table 2; Table 3; Figure 2A–C).

## 4. Discussion

The relationship between BiMax surgeries and the volume of the UA has become a topic of interest recently. There is a spectrum of scientific research reporting the expansion of the UA following this procedure [4,10]. However, in the authors’ opinion, there is an insufficiency of studies that perform the analysis at different reference time points and in a supine position. This fact was the foundation for the creation of this work.

There is a wide range of options concerning the setting of the anatomical and cephalometric constraints of the UA presented in contemporary studies [11]. After reviewing the available literature, we decided to set the boundaries based on cephalometric points and lines (Porion, FH line, PNS) and the lowest point of the epiglottis from a lateral view. We believe that the proposed boundaries are a reliable and fast way of highlighting the UA and can be easily replicated in future studies.

This study was conducted based on the images acquired from a medical CT scanner. The choice of radiological hardware is another major consideration. As mentioned earlier, most studies on this topic are based on CBCT images [12,13]. The use of the mentioned device in oral and maxillofacial surgery settings has expanded rapidly in recent years. When compared with a standard CT scanner, it is notable for its small size, affordability, and reduced ionizing radiation exposure. Its scans are performed in a vertical position and the patient can be easily aligned to their natural head position (NHP) [14]. On the other hand, the conventional CT apparatus provides a much higher soft tissue contrast and resolution with less image noise and artifacts [15]. These factors may relate to the potentially better overall quality of the 3-D renders and analysis of the UA region.

Another noteworthy aspect is the patient setting during the examination. In a study by Cedric S. Van Holsbeke et al., the average value difference of the minimal cross-sectional area of the UA varied greatly depending on the patient position during the image acquisition process (9.76% higher in a standing vs. supine position). The supine position results in a decrease in the UA volume due to the collapse of the overlaying soft tissues [16]. According to Simon A. Joosten et al., the severity of the obstruction of the UA in OSA patients depends on their body position, with the worsening of OSA indicators in up to 60% of the patients when studied lying on their backs. In almost 20% of the patients, the obstruction of the airway happened only in a supine position [17]. Therefore, we believe that radiological examinations performed in a laying position provide more valuable insight into the morphological changes of the UA, particularly in the context described in the present study.

OSA is a sleep-related breathing disorder. It is characterized by respiratory pauses resulting from a partial or complete blockage of the pharyngeal airway lasting at least 10 s. It is estimated that even up to 20% of the global adult population suffers from it, with men being affected more frequently than women [9,18]. The morphology of the UA is only one of the factors leading to OSA’s presence, as it has a multifactorial etiology. Neurological and hormonal pathological changes, obesity, and the improper function of the muscles of the head and neck area add to the spectrum of the pathophysiology of the OSA disorder [19,20]. According to a study by Xing-Long Wen et al., patients with OSA disorder had a notably smaller UA volume [21]. Other studies conclude that an increase in UA volume is correlated with the demise of OSA symptoms [22], and, thus, BiMax advancement surgery is a relevant treatment option for OSA [4].

The present study was performed on patients presenting with dentofacial abnormalities only. The main goal of the described procedures was to restore proper occlusion and facial skeletal relations, not specifically treat the potential OSA resulting from the underlying obstruction of the UA. Despite that fact, the outlined results reveal the significant growth of the UA volume resulting from BiMax advancement.

The authors wish to highlight a potential deficiency in scientific research evaluating the morphology of the UA at various postoperative intervals. The presented results showed a statistically significant progression of the UA volume between the immediate postoperative and 6-month postoperative examinations. This fact could be attributed to the resolution of postoperative edema, the healing of adjacent tissues, and arguably an increase in muscle tension and function due to the restored craniofacial relations and occlusion. Wallace S and McGrath BA [23] reported that laryngeal injury occurs in between 57 and 83% of all patients undergoing prolonged intubation. It can cause local edema, which may lead to respiratory failure and the possibility of reintubation. Despite the unavoidable local irritation and swelling resulting from endotracheal intubation, we did not observe a reduction in UA volume between the preoperative and immediate postoperative mean values. The direct and immediate impact of BiMax advancement surgery may mitigate the early adverse effects of endotracheal intubation on the morphology of the upper airways.

Furthermore, the authors believe that, based on the presented, achieved results and available bibliography, BiMax advancement surgery should always be considered as a potential treatment option for OSA patients [4,12]. A thorough assessment of respiratory system function and a comprehensive evaluation of the morphology of the UA are necessary steps in the pre-surgical assessment conducted by both the orthodontist and the surgeon [24].

Recent advancements and the increasingly widespread accessibility of virtual planning technology should also be discussed and highlighted. Medical professionals, including the surgical team, can visualize the procedure in advance, make a thorough analysis of the patient’s morphology, including the UA, and individualize the surgical plan accordingly, not only to the presented dentofacial abnormality but also to the potential underlying UA obstruction.

The following limitations apply to this study. The constrained sample size of 31 patients included in this study potentially limits its statistical power and should be considered. Only the results presented with a high statistical significance should be relevant to derive any judgements. There is also the problem of the limited control over the data collection process and the associated risks, as well as the exclusion of the patients’ functional outcomes due to the retrospectivity of the study.

The potential selection bias, which may have led to inaccurate and unrepresentative results, was addressed by including all of the BiMax advancement surgeries performed in a presented timeframe with the addition of previously mentioned inclusion criteria; only complete patient data sets were used; all the data included were stored in the same manner on institutional data servers; and the data used came from a specifically defined, recent time period.

The possible measurement bias was addressed by the standardized collection process described in Section 2.3 and Section 2.4 performed on superimposed 3-D models; all the measurements were performed using two-person double checks of the data; and measurements were performed only on complete data sets.

Future prospective studies involving control groups, larger cohorts, and multiple postoperative assessments of the UA, and their correlations with respiratory symptoms, would be advantageous and provide a broader understanding of the surgery’s impact on the UA.

## 5. Conclusions

The significant increase in UA volume observed 6 months postoperatively, compared to the baseline volume, demonstrates the efficacy of BiMax advancement surgery in altering the morphology of the UA. Furthermore, the continued increase in UA volume during the post-surgical observation period indicates that it is not a stable value achieved immediately following the procedure. Therefore, any studies whose results may depend on the volume of the upper airways must be appropriately postponed after surgery.

These presented postoperative findings should influence future studies to include longer follow-up assessments, which could provide more comprehensive data regarding the long-term stability and potential delayed changes in the UA volume.

In addition, surgeons performing orthognathic procedures should always analyze the morphology of the UA and associated comorbidities and provide patients with the possible respiratory benefits resulting from BiMax advancement surgeries. Improvements in the UA volume should be considered as one of the indications for the surgery.

## Figures and Tables

**Table 1:** Patients’ gender and type of skeletal malocclusion.

		Sex		Total
		Female	Male	
Skeletal malocclusion type	Type II	9 (29%)	0 (0%)	9 (29%)
	Type III	15 (48%)	7 (23%)	22 (71%)
Total		24 (77%)	7 (23%)	31(100%)

**Table 2:** Mean and median values of the volume of the UA in selected valuations.

	UA Volume Pre-op (cm^3^)	UA Volume Post-op (cm^3^)	UA Volume 6 m Post-op (cm^3^)
Mean ± SD	22.2 ± 6.4	24.2 ± 7.3	27.3 ± 7.3
Median (Min–Max)	22.8 (9.0–38.2)	22.7 (13.4–41.9)	27.9 (14.1–42.0)
IQR	9.0	11.6	10.0

**Table 3:** The results of *t*-student test for dependent variables.

	Difference of Means	*t*-Test Value for Dependent Variables	*p*	95% CI
Pre-op vs. post-op	−2.1 ± 5.2	−2.18	0.037	(−4.00 to −0.13)
Post-op vs. 6 m post-op	−3.1 ± 4.9	−3.51	0.001	(−4.92 to −1.30)
Pre-op vs. 6 m post-op	−5.2 ± 4.5	−6.46	<0.001	(−6.81 to −3.54)

## Data Availability

Additional study data are openly available at: https://doi.org/10.6084/m9.figshare.26243924, and by the email inquiry of the corresponding author.

## Supplementary Materials

The following supporting information can be downloaded at: https://www.mdpi.com/article/10.3390/jcm13164588/s1, Table S1.
